# Robust tobacco smoking self-report in two cohorts: pregnant women or men and women living with or without HIV

**DOI:** 10.1038/s41598-023-34249-x

**Published:** 2023-05-12

**Authors:** Marie-Soleil R. Smith, Sara Saberi, Abhinav Ajaykumar, Mayanne M. T. Zhu, Izabelle Gadawski, Beheroze Sattha, Evelyn J. Maan, Julie Van Shalkwyk, Chelsea Elwood, Neora Pick, Melanie C. M. Murray, Isabelle Boucoiran, Deborah M. Money, Hélène C. F. Côté

**Affiliations:** 1 Department of Pathology and Laboratory Medicine, University of British Columbia (UBC), Vancouver, BC Canada; 2 Centre for Blood Research, UBC, Vancouver, BC Canada; 3 Women’s Health Research Institute, Vancouver, BC Canada; 4 Oak Tree Clinic, Vancouver, BC Canada; 5 Department of Obstetrics and Gynaecology, UBC, Vancouver, BC Canada; 6 Department of Medicine, Division of Infectious Diseases, UBC, Vancouver, BC Canada; 7 Department of Obstetrics and Gynaecology and School of Public Health, Université de Montréal, Montreal, QC Canada; 8 Women and Children Infectious Diseases Center, CHU Sainte-Justine, Montreal, QC Canada; 9 Department of Pathology & Laboratory Medicine, University of British Columbia, G227-2211 Wesbrook Mall, Vancouver, BC V6T 2B5 Canada

**Keywords:** Biomarkers, Health care, Medical research, Risk factors

## Abstract

Understanding the true burden of tobacco smoking on adverse pregnancy outcomes is critical in generating appropriate interventions to improve outcomes. Self-reporting of human behaviour that is associated with stigma is associated with underreporting in general and may bias the impact of smoking in studies; however, self-reporting is frequently the most practical method of gleaning this information. The objective of this study was to evaluate concordance between self-reported smoking and concentrations of plasma cotinine, a biomarker of smoking, among participants enrolled in two related HIV cohorts. A total of 100 pregnant women (76 living with HIV [LWH] and 24 negative controls) in their third trimester, and 100 men and non-pregnant women (43 LWH and 57 negative controls) were included. Among all participants, 43 pregnant women (49% LWH and 25% negative controls) and 50 men and non-pregnant women (58% LWH and 44% negative controls) were self-reported smokers. The odds of discordance between self-reported smoking and cotinine levels were not significantly different between self-reported smokers and non-smokers, nor between pregnant women and others, but were significantly increased, regardless of self-reported status, among people LWH compared to negative controls. The overall concordance between plasma cotinine and self-reported data among all participants was 94% with a sensitivity and specificity of 90% and 96%, respectively. Taken together, these data demonstrate that participant surveying in a non-judgemental context can lead to accurate and robust self-report smoking data among both persons LWH and not, including in the context of pregnancy.

## Introduction

In 2016, over 1.1 billion people smoked tobacco (21.9% of the global population), varying widely between countries, from 4 to 47%^1^. Approximately one in ten Canadians reported smoking in 2019^2^, which is similar to the United States with 12.5% of adult cigarette smoking in 2020^3^. Rates of smoking are consistently higher among people living with HIV (LWH) than that of the general population. An analysis in the United States with data collected in 2016 noted that the rate of current smoking was twice as high among people LWH (47.0% vs 25.5%)^4^, with similar trends noted in a 2014 Canadian study (~ 30%)^5^. The adverse health effects of tobacco smoking and its association with cancers, cardiovascular disease and premature mortality are well-documented^6^, and are exacerbated in HIV populations in which smoking while LWH reduced life expectancy by > 6 years compared to LWH and not smoking^7^. Smoking is also an established risk factor for adverse pregnancy outcomes, including increasing the likelihood of preterm delivery, intrauterine growth restriction, low birth weight, and infant death^8–12^. The rate of smoking among pregnant women varies world-wide, from 10% in Japan^13^, to 17% in Australia^14^, ~ 20% in Canada^15^, and 30–35% in Spain^16^. Importantly, the effect of smoking on adverse pregnancy outcomes and pregnancy loss in North American women was reported to differ dramatically by HIV status, despite the use of effective antiretroviral therapies^17^.

Most cohort studies collect information on smoking using self-report data; however, depending on the context, the stigma associated with smoking may lead to underreporting and possible bias in studies relying on self-reported data^18–22^. In the non-pregnant population, misclassification is often higher among persons who self-report as non-smoking than the reverse, a phenomenon that appears increased in the context of pregnancy^23^. Indeed, previous studies reported higher non-disclosure rates of smoking among pregnant women than the general population^24–26^. For example, in a smoking cessation trial in the USA, pregnant women underreported their smoking by 14%^24^. Additionally, a study of pregnant women LWH in the United States identified only a weak agreement between self-reported and laboratory confirmed smoking, which the authors attribute to social desirability^27^. Canadian data are scarce and conflicted on the validity of self-reported smoking behaviors in pregnant and non-pregnant populations, and even less is known in the HIV population^15,22,28–32^. In the context of pregnancy, in order to provide optimal care for women, it is particularly important to foster a trusting, non-judgemental environment. This favors the obtention of accurate information by the prenatal care provider on tobacco smoking, as well as other risk behaviours that could adversely impact the health of the mother and the fetus. At the BC Women’s Hospital, and at partner institutions in Canada that provide prenatal care to women living with HIV, women centred non-judgemental trauma informed care is the model utilized, which facilitates disclosure of health behaviours and socio-structural issues for the benefit of the mother and her infant^33,34^.

Cotinine, a nicotine metabolite with a ~ 16 h half-life in plasma, urine, or saliva, is often used as a biomarker of smoking and can be used to determine concordance between levels of the marker and self-reported smoking status^35^. Cotinine has also been identified in cord blood and infant urine as an indicator of fetal exposure to tobacco^36^. In many studies, self-reported smoking is used as a key variable in analysis of clinical outcomes yet has been questioned as a valid reflection of actual smoking rates. Currently, there is a gap in knowledge regarding the validity of self-reported smoking status among pregnant women LWH. The objective of this study was to examine the concordance between self-reported smoking and plasma cotinine concentration among pregnant women LWH and negative controls enrolled in the Canadian CARMA (Children and women, Antiretrovirals and Markers of Aging)-PREG Cohort. Additionally, we examined the concordance between self-reported smoking and plasma cotinine concentration among people LWH and negative control women and men enrolled in the Canadian CARMA-CORE Cohort as a secondary cohort to validate our findings in a similar, non-pregnant, sample.

## Results

In this study, at their study visit taking place between 28 and 38 weeks of gestation, 43 (43%) CARMA-PREG participants self-reported having smoked since last visit (Table 1). In this selected study sample, there was no significant difference in maternal age or HIV status between the self-reported smokers and non-smokers. However, there were fewer Indigenous women in the non-smokers group and fewer African Caribbean Black women in the smoker’s group (p < 0.001). Compared to non-smokers, self-reported smokers were more likely to have a low income (< $15,000/year, p < 0.001), and to use illicit drugs (p < 0.001) (Table 1).

**Table 1:** Demographic, clinical, and substance use characteristics of the study participants from two separate cohorts self-reporting tobacco smoking at their study visit, or not.

	CARMA-PREG			CARMA-CORE		
	Smokers (n = 43)	Non-smokers (n = 57)	p-value	Smokers (n = 50)	Non-smokers (n = 50)	p-value
Age (years)	31 ± 5 (17–42)	33 ± 5 (21–45)	0.138	42 ± 9 (17–75)	38 ± 16 (17–75)	0.132
Weeks of Gestation at Visit^a^	34 ± 2 (30–38)	34 ± 2 (28–38)	0.854			
Female sex	43 (100)	57 (100)	1.000	25 (50)	26 (52)	1.000
Smoking intensity						
Heavy (> 20 cig/day)	3 (7)			7 (14)		
Moderate (2–20 cig/day)	28 (65)			29 (58)		
Light (< 2 cig/day)	3 (7)			11 (22)		
No intensity data	9 (21)			3 (6)		
Study site			0.284			
Vancouver	38 (88)	54 (95)		50 (100)	50 (100)	
Montreal	5 (12)	3 (5)				
Ethnicity			** < 0.001**			** < 0.001**
White	19 (44)	26 (46)		25 (50)	20 (40)	
African Caribbean Black	2 (5)	15 (26)		3 (6)	3 (6)	
Indigenous	19 (44)	4 (7)		20 (40)	8 (16)	
Asian/Other	3 (7)	12 (21)		2 (4)	19 (38)	
Income < $15,000 CAD/year^a^	31 (74)	17 (30)	** < 0.001**	37 (77)	10 (23)	** < 0.001**
HIV + status	37 (86)	39 (68)	0.058	25 (50)	18 (36)	0.225
Substance use						
Illicit drugs^a,b^	23 (54)	4 (7)	** < 0.001**	22 (44)	3 (6)	** < 0.001**
Alcohol	17 (40)	16 (28)	0.284	27 (54)	36 (72)	0.097
Cannabis^a^	9 (21)	6 (11)	0.168	21 (42)	8 (16)	**0.008**

Data are presented as mean ± SD (range) or n (%). Fisher’s exact test and Student’s t-test used.

^a^Data was missing for the following groups CARMA-PREG smokers; CARMA-PREG non-smokers; CARMA-CORE smokers; CARMA-CORE non-smokers for weeks of gestation at visit n = 0; 3; N/A; N/A, income < $15,000 CAD/year n = 1; 1; 2; 6, illicit drug use n = 0; 0; 0; 1, and cannabis use n = 0; 0; 0; 1.

^b^Illicit drugs includes cocaine, heroin, crack, and methamphetamine.

**Figure 1:**
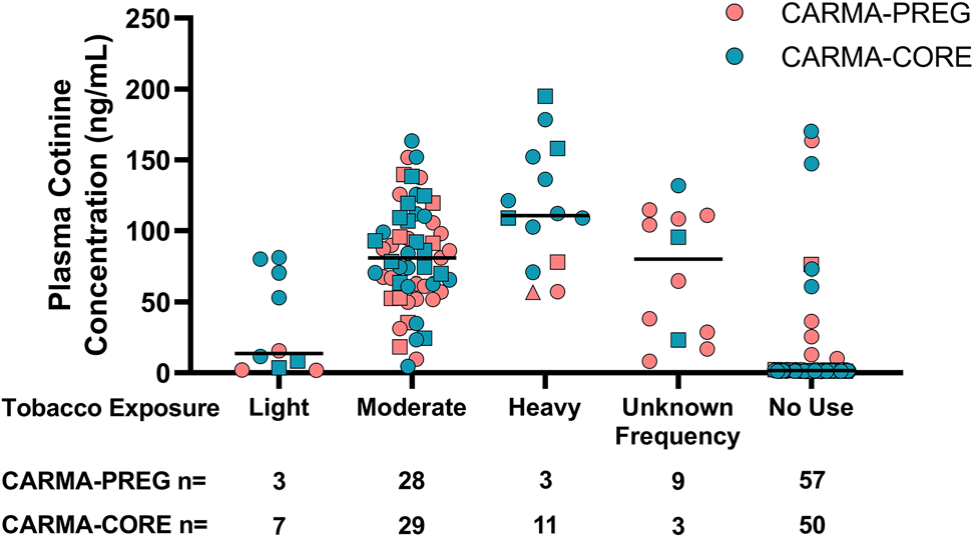
Plasma cotinine concentration according to self-reported intensity of smoking since last visit. Horizontal bar represents median cotinine. The one participant who chewed rather than smoked tobacco is indicated by a triangle, and the participants who used cannabis are indicated by squares.

**Figure 2:**
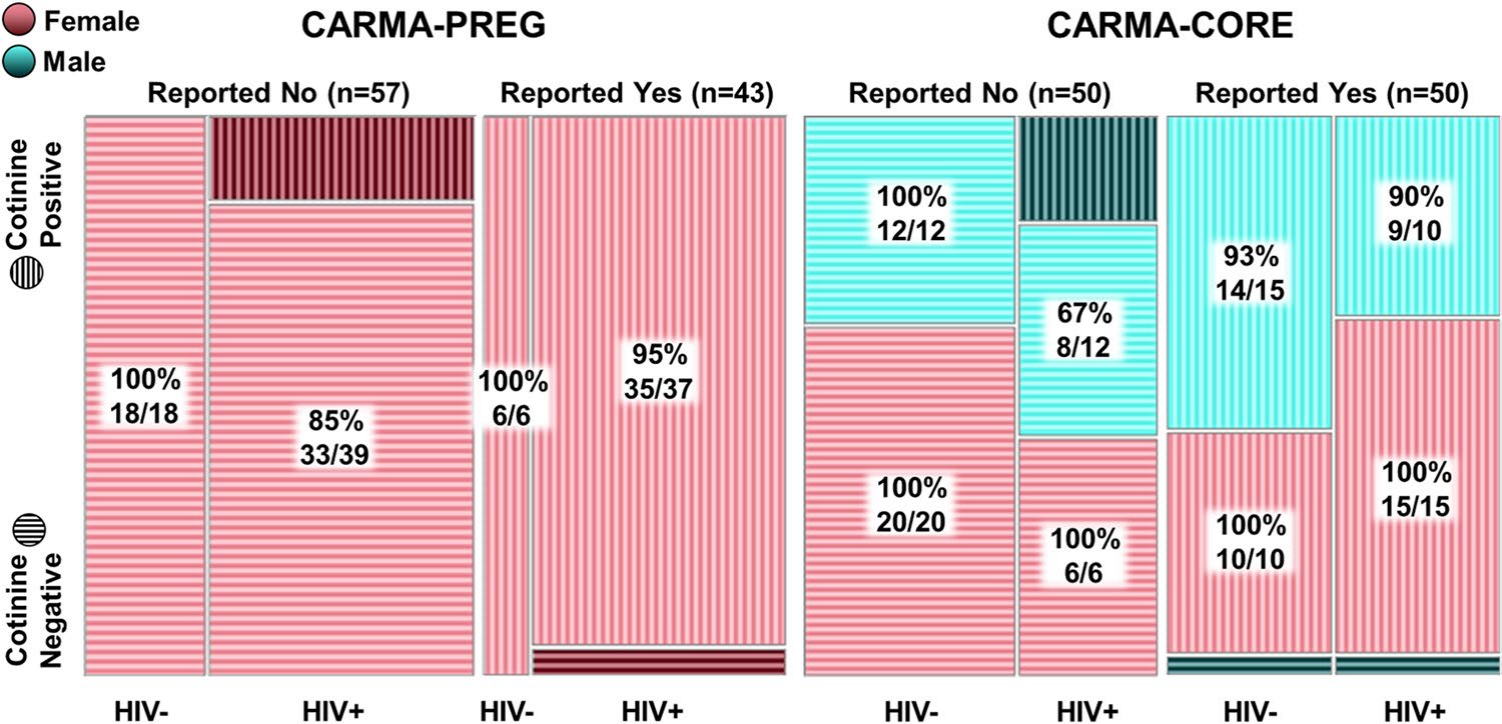
Concordance between self-reported smoking at study visit and plasma cotinine concentration measured by ELISA on a plasma specimen collected on the day of visit in the CARMA-PREG and CARMA-CORE cohorts.

Among pregnant women who reported having smoked (n = 43) since their last visit, 3 (7%) reported smoking heavily (a pack a day or more), 28 (65%) reported smoking a moderate amount (2 to 19 cigarettes a day), and 3 (7%) reported light smoking (less than 2 cigarettes a day) (Figs. 1, S2). The frequency and/or quantity of tobacco use was unavailable for the remaining 9 (21%). Defining plasma cotinine ≥ 5 ng/mL as smoking-positive, we observed 95% concordance between self-reported smoking and smoking positivity according to plasma cotinine. For the women who self-reported as non-smokers, concordance with plasma cotinine < 5 ng/mL was 89% (Fig. 2). Two pregnant women who self-reported smoking showed plasma cotinine levels below 5 ng/mL (Fig. 2). Both women self-reported smoking fewer than two cigarettes per week on average during their pregnancy. The κ for women in CARMA-PREG was 0.839, indicating almost perfect agreement. The sensitivity and specificity of self-reporting in CARMA-PREG were 87% and 96%, respectively. Among pregnant women LWH (n = 76), the concordance between plasma cotinine and self-reported smoking (n = 37) and non-smoking (n = 39) was 95% and 85%, respectively (Fig. 2). Among negative control pregnant women (n = 24), 6 self-reported smoking and the remaining 18 reported no smoking, and the observed concordance was 100%. The κ for pregnant women LWH and negative control pregnant women, were 0.790 and 1.00, respectively. No significant difference in concordance between pregnant women LWH and negative control pregnant women was noted (p = 0.19).

To further examine the quality of self-reported smoking data in our cohorts we examined the concordance of self-reported smoking data and plasma cotinine in the CARMA-CORE cohort. The rate of smoking among participants in the entire cohort for whom this data was available (n = 623) was 40%. Random selection of sex-matched smokers (n = 50) and non-smokers (n = 50) resulted in age and HIV status balanced groups (Table 1), with ages ranging from 17 to 75 years, and both sexes were equally represented. The groups differed significantly by ethnicity, with more Indigenous people in the smoker group and more Asian or other ethnicities in the non-smoker group (p < 0.001). Similar to CARMA-PREG, self-reported smokers were more likely to have low income (p < 0.001) and use illicit drugs (p < 0.001). Additionally, smokers were more likely to use cannabis (p = 0.008).

Among the women and men who reported tobacco use at their first visit in the CARMA-CORE cohort, 11 (22%) reported smoking heavily, 29 (58%) reported smoking a moderate amount, and 7 (14%) reported light smoking (Figs. 1, S2). The intensity of tobacco use was unavailable for the remaining 3 (6%). We observed 96% concordance between self-reported smoking and plasma cotinine. For the women and men who self-reported as non-smokers, concordance with plasma cotinine < 5 ng/mL was 92%. The κ for participants in CARMA-CORE was 0.880. The sensitivity and specificity of self-reporting in CARMA-CORE were 92% and 96%, respectively. Among all CARMA-CORE participants LWH in this study (n = 43), the concordance between plasma cotinine and self-reported smoking (n = 25) and non-smoking (n = 18) was 96% and 78%, respectively (Fig. 2). Among the negative control women and men (n = 57), 25 self-reported smoking and the remaining 32 reported no smoking, and the observed concordance was 96% and 100%, respectively (Fig. 2). The κ for participants LWH and negative controls in CARMA-CORE were 0.755 and 0.964, respectively. No significant difference in concordance between participants LWH and negative controls in CARMA-CORE participants was noted (p = 0.08). Among all female CARMA-CORE participants (n = 51), the concordance between plasma cotinine and self-reported smoking (n = 25) and non-smoking (n = 26) was 100% (Fig. 2). Male CARMA-CORE participants (n = 49) had a concordance between plasma cotinine and self-reported smoking (n = 25) and non-smoking (n = 24) of 92% and 83%, respectively (Fig. 2). The κ for female and male CARMA-CORE participants were 1.00 and 0.755, respectively. There was a significant difference in concordance between male and female CARMA-CORE participants, in which men were more likely to have a discordant self-report (p = 0.01).

Taken together, if we group the two datasets, among 100 pregnant women, 51 non-pregnant women, and 49 men included in our analyses, 93 (47%) self-reported smoking. We observed an overall concordance of 94% between plasma cotinine and both self-reported smoking and non-smoking data with a κ of 0.860 and the sensitivity and specificity were 90% and 96%, respectively. Among all individuals LWH in both cohorts (n = 119), the concordance between plasma cotinine and self-reported smoking (n = 62) and non-smoking (n = 57) was 95% and 81%, respectively. Among all individuals who did not have an HIV diagnosis (n = 81), 31 self-reported smoking and the remaining 50 reported no smoking, with an observed concordance of 98% and 100% respectively. The κ coefficients for individuals LWH and negative controls were 0.780 and 0.974 respectively and although both high, these were significantly different (p = 0.009). It is notable that discordance was present for both self-reported smokers and non-smokers, indicating a bidirectional discordance among the HIV group. Among all women in both cohorts (n = 151), the concordance between plasma cotinine and self-reported smoking (n = 68) and non-smoking (n = 83) was 98% and 95%, respectively, with a κ of 0.894. There was no difference in concordance between people who used or did not use cannabis (p = 0.74).

For seven CARMA-PREG participants who self-reported smoking and had high plasma cotinine concentration (> 75 ng/mL), and seven participants for whom self-report and cotinine were discordant, we investigated the relationship between cotinine concentrations at third visit, at delivery, and in cord plasma. Participants with high cotinine levels at third visit also showed high levels of cotinine in maternal and cord plasma at delivery (Fig. S3). Two of the seven participants with discordant self-report and cotinine values reported light smoking but consistently had cotinine values below the 5 ng/mL limit (Fig. S4). Of the five remaining discordant participants who self-reported non-smoking but had cotinine values > 5 ng/mL at third visit, three had cotinine values < 5 ng/mL at delivery in maternal and cord plasma and two remained > 5 ng/mL throughout (Fig. S4).

For quality control purposes, we repeated the ELISA on 74 of the CARMA-PREG specimens. Qualitatively, we obtained identical results, indicating the assay is robust enough to assay specimens only once.

## Discussion

The prevalence of maternal smoking in the entire CARMA-PREG cohort (28%) is higher than the estimated rate of smoking during pregnancy in Canada^11,28,37^, and is likely related to the demographic and socioeconomic makeup of our cohort. Of note, prevalence of smoking was similar (30%) in participants who were not included in the final analysis, indicating that bias was not introduced through the exclusion criteria. Although we purposefully selected similar numbers of smokers and non-smokers from the CARMA-CORE cohort for cotinine analysis, the rate of smoking in the entire cohort was 40%, four times as high as the Canadian average, but similar to reported rates among persons LWH in Canada^2^. Additionally, our results are consistent with previous Canadian studies showing that low income is associated with higher smoking rates^38,39^, including during pregnancy^15,28,37^.

As in many studies, the CARMA cohorts record smoking data via self-report, collected through interviews with research staff. We undertook the current study to examine the concordance between self-report and a biomarker of smoking, namely plasma cotinine, to ascertain whether self-reported smoking was a reliable variable for biomedical analyses in our studies. This was sparked, in part, by consistent observations of tobacco smoking exerting significant effects on the markers of cellular aging we are studying. Of note, people LWH worldwide are more likely to be smokers than people without HIV, implicating smoking as an important covariate in HIV studies^40^. We therefore felt a need to assess the robustness of our data. In participants from two separate cohorts with different research staff, we observed excellent concordance between self-report and cotinine, both among participants LWH and negative controls and in the pregnancy and non-pregnancy cohorts. Additionally, we observed a positive trend between self-reported smoking intensity and plasma cotinine concentration, indicating that the intensity of tobacco use was also accurately reported. Overall, only 9% of self-reported non-smokers were likely to be a smoker considering their cotinine result, and 4% of self-reported light smokers showed plasma cotinine levels ≤ 5 ng/mL. Given the half-life of cotinine, the timing of sampling could influence its detection. Hence, lower concordance between cotinine and self-reported smoking may be expected using this assay when the smoking frequency is very low, especially since its metabolism and clearance may be accelerated during pregnancy^41^.

The majority of the discordant results in this study were observed among people LWH. It is possible that this may in part be related to a reportedly faster nicotine metabolism in people LWH, which can further be affected by the antiretrovirals^42–45^. Given the high prevalence of smoking in some communities, this may also be related to a higher likelihood of second-hand smoke exposure. Additionally, men were more likely to be discordant, an interested finding that could warrant further research.

Our cohort participants were questioned on the use of cannabis and illicit drugs, topics that are sensitive to discuss, especially during pregnancy. Of note, the use of cannabis was not associated with self-reported smoking discordance, with only one discordant participant who self-reported as a non-smoker and reported cannabis use. This suggests that practices that mix tobacco with cannabis were either not performed, or were self-reported as a tobacco exposure.

Our results are similar to a Swedish study reporting that 6% of self-reported non-smoker pregnant women were more likely to be smokers and that 3% had cotinine concentrations suggestive of passive smoking^46^. However, the majority of studies report lower concordance. For example, in West Scotland, 25% of pregnant women who self-reported to be non-smokers had cotinine measurements above the threshold^23^. Similarly, in rural and small metropolitan areas in upstate New York, 35% of pregnant women had urinary cotinine levels suggesting inaccurate self-reported status^19^. In a systematic review conducted in 2009, 40 of the 54 included studies that compared prevalence estimates of self-reported smoking versus measured cotinine values in adults indicated that actual smoking status was underrepresented by self-reporting^47^. Such cases of underreporting may introduce bias to the associated analyses and may have implications not only in the research setting but also for clinicians and caregivers^20^. The integrity of self-reported data varies according to population and the social context in which the data are collected. Multiple factors could influence the interviewee’s responses to questions about smoking status, including participant characteristics, study method and setting, and the pressure mediated by social desirability. The present study was not designed to investigate how these factors may influence or predict the quality of self-reporting.

A strength of this study is that the participants were not aware that their self-reported smoking status would be confirmed via biochemical testing, thus a large majority of participants accurately reported their status without pressure to do so. Additionally, given that 98% of women living with HIV in Canada access ART (hence care) during pregnancy^48^, and that the vast majority of women invited to participate consented to do so, it is unlikely that recruitment bias exerts a large effect on our estimates. Another strength of this study is the high smoking rate within the cohorts, among both participants LWH and negative controls and the multi-ethnic makeup. As this study was conducted by research staff and participants were told the information they provided was confidential, the findings may not be generalizable to how women would share smoking information with their care providers. Although the cohort may not be fully generalizable to all pregnant and non-pregnant populations the goal of this study was to evaluate self-report concordance in the context of research, namely a prospective observational cohort study, and our results show high concordance among all participants. A limitation to this study is that data on exposure to second-hand smoke were not collected, an important factor that may help explain some of our discordant results among self-reported non-smokers.

Overall, our data suggest that it is possible to obtain robust data on smoking from pregnant and non-pregnant participants, living with HIV and not, through self-report in a research setting. Among CARMA cohort participants, our results indicate that self-reported smoking data are highly reliable as a surrogate for tobacco exposure. This suggests that study participants likely felt safe to speak candidly, and accurately self-reported their smoking habits to our non-judgemental research staff who explained during consent that all information is confidential, including from their care providers. This study will allow researchers utilizing this and similar datasets to have higher confidence when using self-reported smoking values in statistical modelling. The validity of this data is of particular importance as clinical studies, which rely on these types of self-reported data, help inform further clinical research.

## Methods

### Study sample

This is a cross-sectional study using data and specimens from two cohorts: CARMA-PREG and CARMA-CORE. CARMA-PREG is a prospective cohort which enrolled pregnant women LWH and negative controls between 2004 and 2020. Pregnant women LWH were recruited at the Oak Tree Clinic in British Columbia (BC) Women’s Hospital in Vancouver, BC, and the Sainte-Justine Hospital in Montreal, Quebec. The Oak Tree Clinic utilizes a model of women-centered HIV care to provide multiple services, including harm reduction and addition counselling by care providers, for women LWH^33^. Negative control women were recruited at BC Women’s Hospital. The enrolment of women in CARMA-PREG took place during their first prenatal visit and both women LWH and negative controls provided biological specimens at three visits during pregnancy, at delivery, and post-partum. Cord blood was also collected. Inclusion criteria for enrolment in the CARMA-PREG cohort were being pregnant with a known HIV status, and women LWH had to be receiving or be willing to receive antiretroviral therapy during pregnancy. Exclusion criteria for the cohorts included the inability to provide informed consent (language barriers) or to participate in research (health or social crisis). Additional exclusion criteria for the selection of the study sample for this sub-study of CARMA-PREG included the exclusion of participants missing third trimester plasma specimens, between 28 and 38 weeks of gestation, and the exclusion of those missing smoking information. The 167 participants excluded due to missing third trimester specimens included 109 from which the specimen was empty due to use in previous studies, 22 specimens were of too low volume for the assay, 15 were missing due to preterm birth (third trimester visit did not take place), and 21 were missing from the inventory. Despite having access to specimens from other trimesters, we chose to only include third trimester specimens in which the participants were all in the same period of pregnancy and equally visibly pregnant, a time in which stigma may be highest. For CARMA-PREG participants with repeat pregnancies, only the first was considered, and if a participant was enrolled in both cohorts, the CARMA-PREG data was prioritized. See Fig. S1 for details on the study participant selection.

Enrolment of participants LWH in CARMA-CORE took place during routine clinical visits at Oak Tree Clinic. Negative control participants were recruited through a variety of means, including word of mouth and advertisements posted in strategic areas in Vancouver to promote enrollment of negative control participants with similar sociodemographic characteristics to participants LWH, including smoking habits. Recruitment took place between 2008 and 2017 and participants had between one and seven study visits during that period. Inclusion criteria for enrolment in the CARMA-CORE cohort were a known HIV status, and the ability to provide informed consent. For the selection of the study sample for this sub-study, those younger than 14 years of age, missing plasma specimens from the first visit, or missing smoking information were excluded. Those younger than 14 years of age were not included as information on their smoking status was not collected. If a participant preferred not to answer, or their smoking status was unknown, then their smoking status was recorded as missing data.

Among all participants eligible for inclusion in this sub-study, 57 non-smoking CARMA-PREG participants were selected to match year of visit with the remaining n = 43 smokers, for a total of 100 participants. Smokers and non-smokers in CARMA-CORE were sex matched and 50 in each group were randomly selected from the whole cohort for a total of 100 participants.

For both cohorts, demographic data were collected upon entrance to the study, and clinical and substance use information, including tobacco exposure, were collected by self-report (any tobacco use since last visit) at each visit through participant interviews with trained research staff embedded in the clinical setting. For 81 of the 93 (87%) self-reported smokers, the intensity of tobacco smoking was collected and later categorized as heavy smoking (a pack a day or more), moderate smoking (2 to 19 cigarettes a day), or light smoking (less than 2 cigarettes a day). Smoking intensity definitions were adapted from the Government of Canada tobacco use statistics^49^. Research staff were instructed to not react or pass judgement during the survey, to ensure a safe research environment. In accordance with this, the research staff did not provide any recommendations for smoking cessation. Participants were also informed that their information would be confidential and would not be shared, even with their treating physician who was an investigator on the study. At the time of data collection, no self-report around tobacco exposure via vaping was sought as it was exceedingly rare, if occurring at all. For one participant, information around tobacco chewing (1 pinch 5 ×/day) was collected and converted to interpret one pinch as equivalent to 4 cigarettes^50^. No data were ever collected about second-hand smoking, nicotine patch, or nicotine gum use. Participants were not informed that cotinine would be measured, thus data were anonymized for these analyses.

### Plasma cotinine measurement

Plasma cotinine levels were measured in specimens collected at their third trimester pregnancy visit from a total of 76 pregnant women LWH and 24 negative control CARMA-PREG participants. Additional plasma specimens at delivery and cord blood from select participants, 7 with high cotinine values and 7 with discordant self-report and cotinine values, were also assessed. In the CARMA-CORE cohort, plasma cotinine levels were measured in specimens collected during their first visit from a total of 43 people LWH and 57 negative control participants. Self-reported substance use information was collected on the same day as blood collection. Cotinine was measured by solid phase competitive enzyme-linked immunosorbent assay (ELISA) (Calbiotech, CA, USA). Any cotinine ≥ 5 ng/mL was defined as cotinine-positive, based on the limit of detection of the assay.

### Statistical analyses

Comparisons between self-reported smoker and non-smoker groups were done using Fisher's exact test for categorical variables and Student t-test for continuous variables. The proportions of cotinine-negative among self-reported non-users and of cotinine-positive among self-reported users were used to express concordance. In addition, Cohen's kappa coefficient (κ), sensitivity, and specificity were used to determine the agreement between smoking as per self-report and cotinine among all participants and within the LWH and negative control groups. The association between the self-reported number of cigarettes per day and plasma cotinine levels were explored through Pearson’s correlation. All statistical analyses were performed using GraphPad Prism 8.4.3 and JMP Pro 15.

### Ethics declarations

Ethical approval for this secondary use of data study was obtained from the Research Ethics Boards of the University of British Columbia and from the Hospital Research Review Committee of the Children’s and Women’s Health Centre of British Columbia (H03-70356, H04-70540, H07-03136, and H08-02018). All methods were performed in accordance with relevant guidelines and regulations. All participants provided written informed consent to have their cohort specimens biobanked.

## Supplementary Information

Supplementary Information.

## Data Availability

All study data are included in the article and/or supporting information. All requests for or questions about the data can be initiated by contacting helene.cote@ubc.ca.
