# Supplementary material for: Robust tobacco smoking self-report in two cohorts: pregnant women or men and women living with or without HIV
Source: Sci Rep. 2023 May 12;13:7711. doi: 10.1038/s41598-023-34249-x (PMC10182043; doi:10.1038/s41598-023-34249-x)
Supplement: Supplementary file 1 — Supplementary Information. [file 41598_2023_34249_MOESM1_ESM.docx]

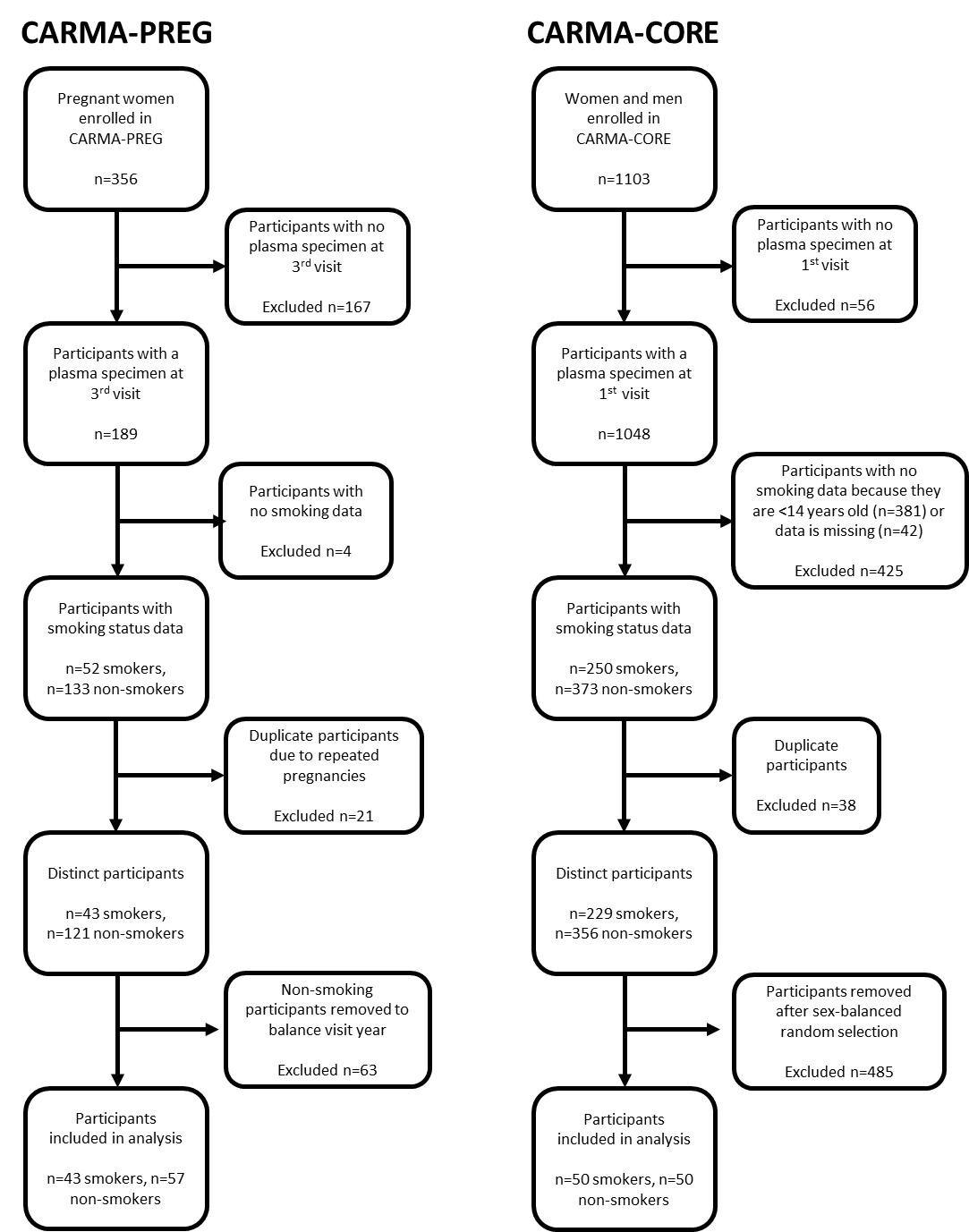


**Figure S1.** Selection criteria for both cohorts.


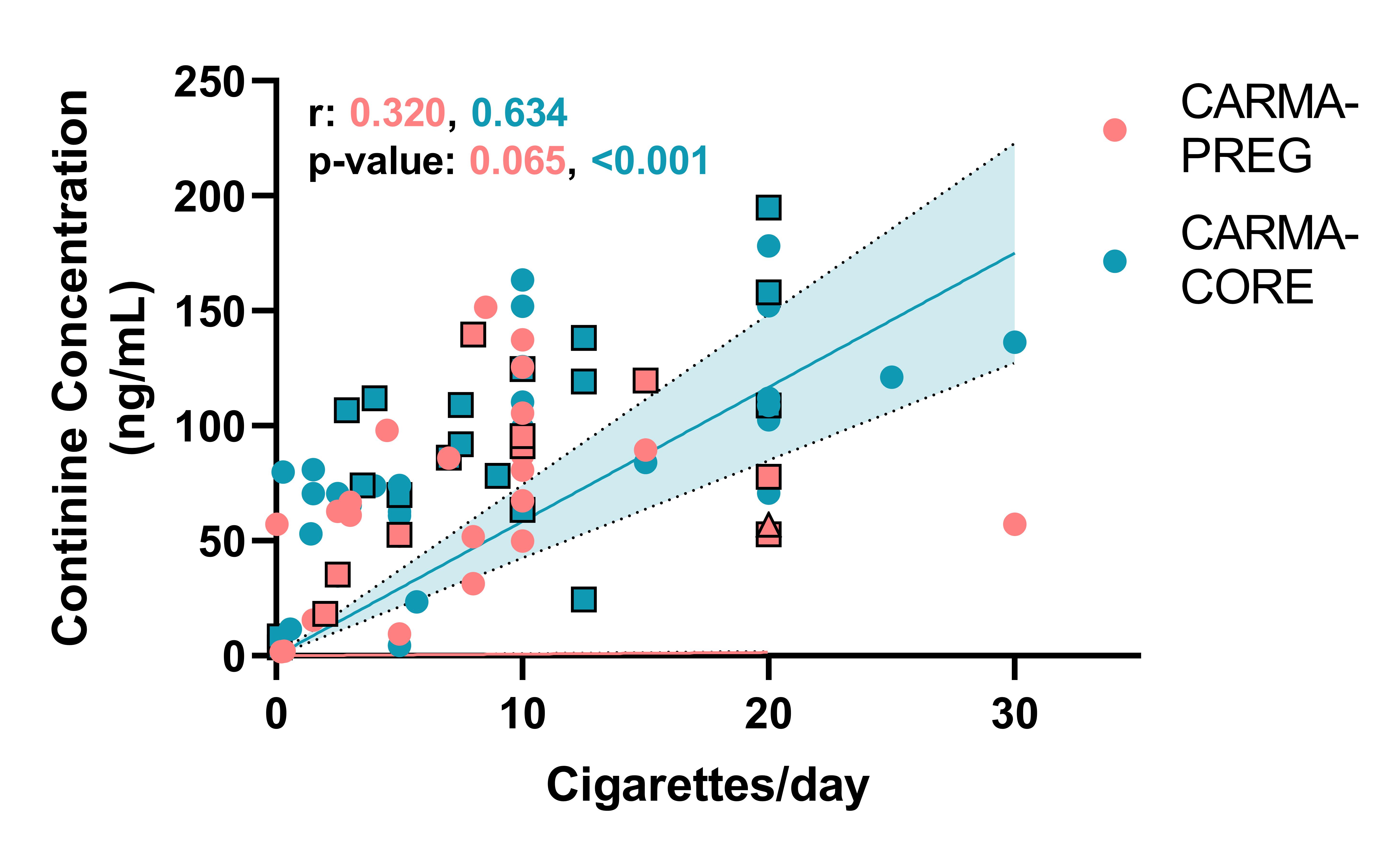


**Figure S2**. Correlation between cotinine concentrations and cigarettes per day from n=34 CARMA-PREG and n=47 CARMA-CORE participants with information on smoking intensity. The one participant who chewed rather than smoked tobacco is indicated by a triangle, and the participants who used cannabis are indicated by squares.


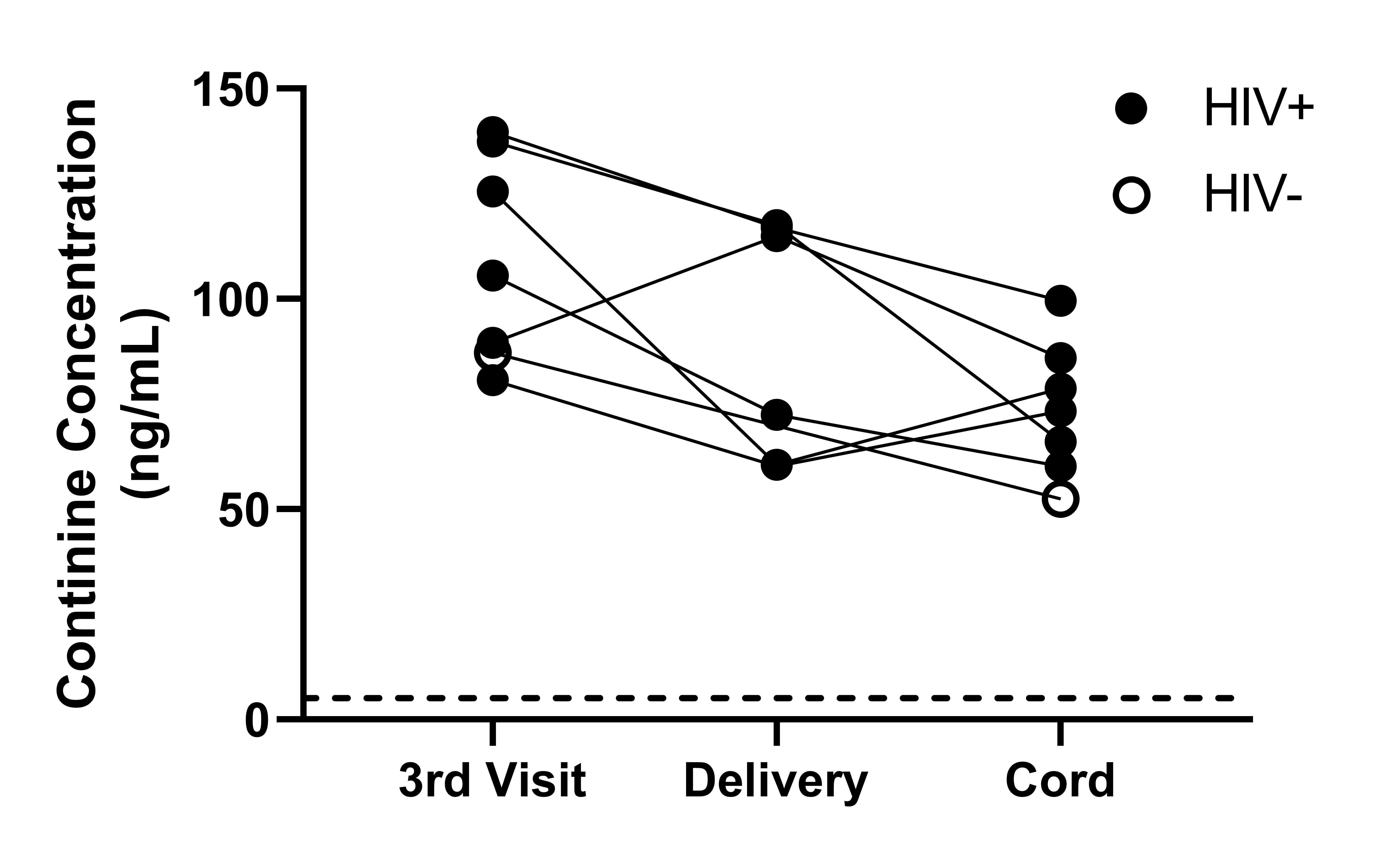


**Figure S3.** Cotinine concentrations from 7 self-reported smokers in CARMA-PREG with high cotinine in plasma at third trimester visit and plasma (n=6) and cord plasma (n=7) at delivery. Dotted line at y=5 indicates cotinine positivity.


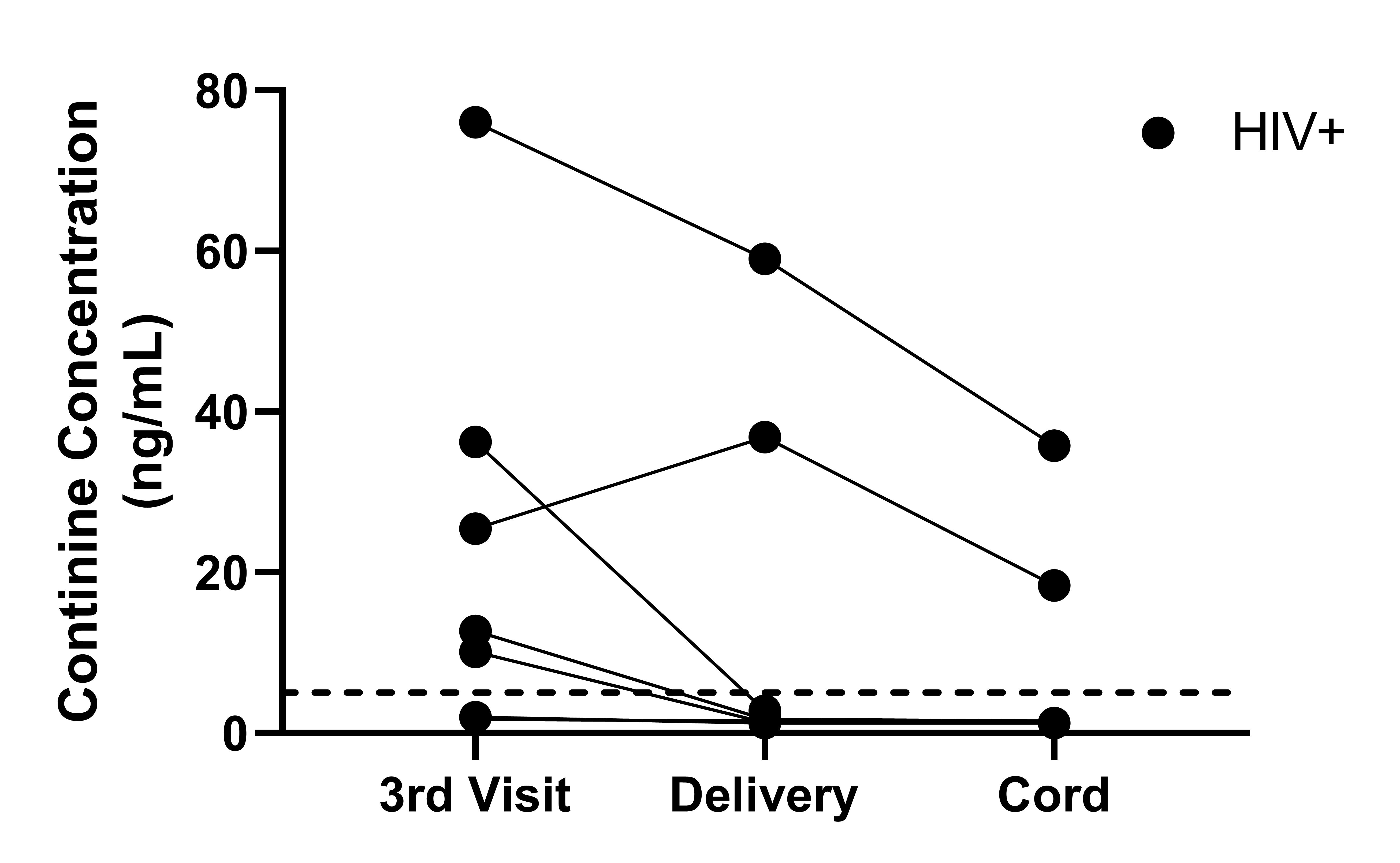


**Figure S4.** Cotinine concentrations from 7 discordant self-report participants in CARMA-PREG in plasma at 3^rd^ visit and plasma (n=6) and cord plasma (n=6) at delivery. Dotted line at y=5 indicates cotinine positivity.
